# Do 12-Week Yoga Program Influence Respiratory Function of Elderly Women?

**DOI:** 10.2478/hukin-2014-0103

**Published:** 2014-11-12

**Authors:** Lídia Aguiar Bezerra, Helton Fabrício de Melo, Ana Paula Garay, Victor Machado Reis, Felipe José Aidar, Ana Rita Bodas, Nuno Domingos Garrido, Ricardo Jacó de Oliveira

**Affiliations:** 1 University of Brasilia, Brasilia, Brazil.; 2 Department of Sport Sciences, University of Trás-os-Montes and Alto Douro, Vila Real, Portugal.; 3 Research Centre for Sport, Health and Human Development, Vila Real, Portugal.; 4 Fire Brigade of Minas Gerais, 5th Battalion Fire Military Fire Brigade of the State of Minas Gerais, New Horizons Program, Uberlandia, Minas Gerais, Brazil.

**Keywords:** elderly, women, respiratory strength, respiratory volumes, yoga

## Abstract

Aging produces several respiratory limitations and reduces tolerance to physical efforts, sometimes leading to pulmonary diseases in the elderly. The literature draws attention to the possible benefits of Yoga practice among the elderly, presenting evidence for significant improvements in quality of life. It was hypothesized that yoga practice can improve respiratory function in the elderly. The effects of a yoga program on pulmonary volumes and respiratory muscle strength were verified in 36 elderly women divided into a yoga group [YG] (63.1 ± 13.3 years of age) and a control group (61.0 ± 6.9 years of age). Maximal inspiratory and expiratory pressure (MIP and MEP) were assessed by a manovacuometer and tidal volume (VT), vital capacity (VC) and minute ventilation (VE) were measured by a ventilometer. The program comprised 65 min sessions, 3 times/week during 12 weeks. The heart rate and respiratory rate decreased significantly in the YG (76-39 ± 8-03 vs. 74-61±10.26 bpm and 18.61 ± 3.15 vs. 16.72 ± 3.12 resp/min, respectively). In the YG, VT and VE increased significantly (0.55 ± 0.22 vs. 0.64 ± 0.2 ml and 9.19 ± 2.39 vs. 10.05 ± 2.11 ml, respectively), as well as VC (1.48 ± 0.45 vs. 2.03 ± 0.72 ml). Improvements were also found in MIP and MEP in the YG (62.17 ± 14.77 vs. 73.06 ± 20.16 cmH2O and 80.56 ± 23.94 vs. 86.39 ± 20.16 cmH2O, respectively). It was concluded that a 12-week yoga program significantly improves pulmonary function of aged women.

## Introduction

Aging produces several respiratory limitations and reduces tolerance to physical efforts, sometimes leading to pulmonary diseases in the elderly (Grinton, 1994; Turcic et al., 2002; Marek et al., 2011). More specifically, aging promotes the reduction of respiratory function (namely pulmonary volumes and respiratory muscle strength) due to gradual loss of pulmonary spring and to reduction of the alveolar area, at the rate of 4% per decade after the age of 30, as well as a reduction in movement amplitude of intervertebral joints (Rossi et al., 1996; Becklake et al., 1999).

Additionally, the aging process is associated with muscle weakness and alterations in pulmonary parenchyma which in turn, affects the ability to generate a satisfactory airflow (Brito et al., 2009; Freitas et al., 2010).

According to Higgibothan et al. (1986) and Rode and Shephard (1994), the loss of pulmonary power together with physical inactivity cause a decrease in the use of peripheral oxygen. Moreover, physical inactivity is related with a decrease of the forced expiratory volume during the first second in men and women (47–74 years old), being a risk factor for cardiovascular diseases, thrombosis and lung cancer (Jakes et al., 2002). Muscle strength in inspiration also decreases both in men and women after 55 years old (Black et al., 1969). This effect may be a consequence of increased flexibility of the muscles responsible for inspiration, as previously observed (Teodori et al., 2003).

Physical exercises increase pulmonary capacity preventing its loss with aging (Babb et al., 1997; Delorey et al., 1999). In case of middle-aged subjects, Pelkonen et al. (2003) showed that physical exercise delayed a decrease of pulmonary function and caused changes in forced vital capacity and other pulmonary volumes.

Among the different types of physical exercise, yoga is becoming more and more important in western civilizations. This is an Indian millenary technique which is aimed at flexibility, muscle strength and meditation, and it includes respiratory exercises which enhance pulmonary capacities (Gopal et al., 1973; Ray and Sinha, 2001; Bernardi et al., 2002; Manocha et al., 2002; Cooper et al., 2003; Mandanmoha et al., 2003). However, the majority of those studies analysed young adults and children, especially those suffering from asthma.

The literature draws attention to the possible benefits of Yoga practice among the elderly, presenting evidence for significant improvements in quality of life (Oken et al., 2006) and mental depression (Krishnamurthy and Telles, 2007). However, the literature is scarce on the effects of yoga exercises on ventilation capacities and respiratory muscle strength in the elderly in a shorter period of time (Vyas and Dikshit, 2002).

Therefore, this study tested the hypothesis that yoga practice can improve respiratory function in the elderly.

## Material and Methods

### Subjects

The sample comprised 36 women randomly divided into two groups of equal size (n=18): a yoga group (YG) with a mean age of 63.1 ± 13.3 years and a control group (CG) with a mean age of 61.0 ± 6.9 years. All the volunteers were residing in Brasília, and surrounding areas of Distrito Federal and the recruitment procedure was performed at the convenience of the researcher. Only women over 55 years of age, not engaged in any type of physical exercise (including yoga) in the previous six months, presenting a functional teeth set and without former pulmonary disease were allowed to participate in the study. All subjects provided their written informed consent to participate in the experiment and the procedures were approved by the institutional Ethics Committee of the Catholic University of Brasília.

### Procedures

The aim of the present study was to analyse the influence of a 12-week yoga program on pulmonary volumes and respiratory muscle strength in elderly women.

Before the start of the experiment, all women went to the Studies in Physical Exercise and Health Laboratory (Brasília Catholic University-Brazil) to discard any possible medical counter indication. Their resting peripheral oxygen saturation and cardiac frequency were assessed with a *Nonin*^®^ oximeter. Resting arterial blood pressure measurements were also executed with the use of a *Becton Dickinson*^®^ sphygmomanometer and a *Littmann*^®^ stethoscope. The subjects were submitted to the same measurements one week after the experiment due the laboratory date availability.

In the week prior to the experiment, respiratory muscle strength and pulmonary volume analyses were performed. Before those analyses each subject visited the laboratory for a general explanation of the assessment training protocol (Black and Hyatt, 1969; Volianitis et al., 2001). The subjects then underwent a 12-week yoga exercise program. The program was comprised of three sessions per week, with 65 minute duration of each session. One week after the conclusion of the program the same measurements were taken.

### Respiratory muscle strength assessment

Maximum inspiration pressure (MIP) and maximum expiration pressure (MEP) were measured with an aneroid class B model manuvacuometer (Support^®^), capable of measuring negative and positive pressures with a −160 to +20 cmH_2_O range. The manuvacuometer was connected to a semi flexible cylindrical plastic tube (140 mm width and 25 mm in diameter) where a nozzle and a rigid plastic connector of 1 mm hole were fastened.

### Pulmonary volume assessment

The tidal volume (V_T_), minute ventilation (V_E_) and vital capacity (VC) were assessed with a 70 × 60 mm ventilometer (*Wright Respirometer Mark 8*) attached by a 22 mm connection to a semi flexible cylindrical plastic tube (140 mm width and 25 mm in diameter) where a nozzle and a rigid plastic connector were fastened.

### Evaluation Protocol

All measurements were performed by the same experienced technician. The volunteers remained seated, with a 90° angle between the trunk and the thighs, feet relaxed and one of the arms resting on the thigh (Volianitis et al., 2001). During measurements, verbal encouragement was provided (Volianitis et al., 2001).

Firstly, respiratory volumes were assessed, followed by the MIP and MEP assessment (Clanton and Diaz, 1995).

To take MIP and MEP the participant put the tube against the face and obstructed the nose with a clip and adjusted the teeth to the nozzle which involved also the lips, so that no air escaped. The connector with a 1 mm hole was used to avoid the interference of facial muscles in pressure values (Clanton and Diaz, 1995; Harik-Khan and Wise, 1998; Volianitis et al., 2001). For MIP measures, the participants were told to slowly and completely exhale the air till the residual volume, so that the maximum inspiration effort should be achieved (Black and Hyatt, 1969). For MEP, the subject inspired till the total pulmonary capacity. Then the subject executed a maximum expiratory effort against the closed tube (Black and Hyatt, 1969; Enright et al., 1994; Harik-Khan and Wise, 1998; Volianitis et al., 2001). Both manoeuvers were executed during 2 seconds. At least three trials were performed with two minutes of recovery between them. The highest obtained values were used in the analysis. A five minute rest period was applied between MIP and MEP measurements (Enright et al., 1994). The higher record of MIP and MEP was considered for each subject (Volianitis et al., 2001), as long as the difference between the highest record and the other measurements was less than 10%.

As for V_E_, V_T_ and slow vital VC assessment the volunteers put a nose clip, adjusted the nozzle to the teeth, and closed the lips so that no air escaped. At the end of each evaluation, the volumetric scale was adjusted to zero. Each participant was asked to breathe normally when taking the V_T_ and rate (RR) values. Thus, the V_T_ was calculated through the relationship between the V_E_ and RF. For slow VC, the subject was asked to make a maximum inspiration and next slowly expire till residual volume.

### Heart Rate Assessment

The heart rate (HR) was obtained by a Finger-Tip oximeter (NONIN^®^).

### Yoga program

The yoga program for elderly people lasted 12 weeks and it was performed three times per week, 65 min per session with each yoga exercise session divided into 3 parts: preparatory, main and final (Luby, 1977). Each phase is described in detail below.

Preparatory phase: 5 min relaxing with nasal respiratory exercises and 5 min for a joint warm-up. During respiratory exercises the participants were sitting and performed the following exercises: i) *Adhama Pranayama* (deep breathes with or without air retention); ii) *Kapalabhati* (exhaling the air with vigour through the nostrils); iii) *Nadi Sodhana* (alternated breathing through the nostrils); iv) *Bastrika* (inhaling and exhaling as fast and strong as possible, producing a noise as loud as the sound of a sickle). Concomitantly with these respiratory exercises, techniques for abdominal muscles were also performed: *Udyana Bhanda* (contraction of the abdominal region breathing normally or after a forced exhalation) and *Jalandhara Bandha* (contraction of larynx muscles after an inhalation).

Main phase: 50 min to repeat 15 positions (*asanas),* with 15 to 20 s per each repetition. The positions were as follows: tree position (*vrikasana),* triangle position (*triconasana),* royal ballerina (*natarajasana*), hands on feet *(padahastasana),* wheel position (*chakrasana*), vertebral column torsion (*vakrasana*), incan position *(pashimotanasana)*, snake position (*bhujangasana)*, turtle position (*kurmasana*), cat position (*katuspadasana*), grasshopper position (*salabhasana*), head on the knees (*janusirsharsana*), arch position (danurasana), sail (*sarvangasana)* and the sickle (*viparitakarani*) (Luby, 1977).

Final phase: 10 min for final induced relaxation, with natural and spontaneous breathing through the nostrils with the individuals in dorsal decubitus and eyes closed.

### Statistical Analysis

The results are presented as means and standard deviations. Comparisons between measures were performed with the Split-plot Anova after the normality assumption was confirmed. The significance threshold was set at *p*≤ 0.05.

## Results

Morphological and resting physiological measurements are presented in Table 1. Initial values (pre) did not differ between the two groups. The heart rate decreased significantly in the yoga group (*p*≤ 0.05) but not in the control group. The respiratory rate decreased significantly (*p*≤ 0.05) in the Yoga group between pre and post tests.

Respiratory muscle strength and pulmonary volume measures are presented in Table 2. Initial values (pre) did not differ between the two groups. The yoga program induced significant changes in V_E_, VC, MIP and MEP (*p*≤ 0.05). Post-training V_E_ and VC were higher in the experimental group when compared with the controls (*p*≤ 0.05).

## Discussion

This study tested the hypothesis that yoga practice can improve respiratory function in the elderly. The main finding of this study was that the subjects that were submitted to the yoga program achieved significant changes in their pulmonary volumes and respiratory muscle strength. Additionally, the resting heart rate and respiratory rate also decreased significantly after the 12-week program.

The significant decrease in the heart rate and respiratory rate in the experimental group occurred probably due to an increase of the parasympathetic activity. Indeed, this effect had been previously observed in young adults (Gopal et al., 1973) and in subjects whose age ranged from 20 to 46 years (Vempati and Telles, 2002). This possible mechanism of adaptation was also referred by Bernardi et al. (2002) who observed that baroreflex sensitivity could be reached through slow breathing. This effect may be possible for both healthy people and those suffering from chronic cardiac insufficiency. Probably, the increase of vagal activity and the reduction of sympathetic activity may reduce the HR and RR observed during slow breathing, and it is associated with yoga breathing which substantially reduces the chemo reflex response to hypoxia, probably through the improved oxygen delivery to tissues (Aktar et al., 2013). Other references in the literature also provide data to support practice of yoga to promote a reduction of the HR and RR (Cebriá et al., 2013; Vyas and Dikshit, 2002). Moreover, other than the meditation and relaxation techniques, the subjects in the present study were submitted to a program which also included static positions that could be viewed as a physically demanding exercise. Therefore, the combination of these two elements together may justify the decrease in RF and CF.

In the yoga group, we also observed an increase in the VC, V_E_ and V_T_. In our opinion, these adaptations could be a result of the inclusion of *pranayamas* (respiratory technique) together with the *asanas* (static physical positions) in the yoga program. The *pranayamas* execution covers several percentiles of the total pulmonary capacity and it influences the action of the diaphragm by the quantity of the inhaled air. For instance, the sub maximum activation of the diaphragm within low pulmonary volumes, may be a consequence of the differences of the relation width-inspiration muscles tension and/the inhibition reflex of the frenic moto neuron (McKenzie et al., 1996; Weiner et al., 2003). Additionally, the *pranayamas* also aims to address the relationship between the width-muscle tension (pre and post charge), the elastic pulmonary properties and the different time constants of pulmonary insufflations, as well as the regional difference of perfusion ventilation due to upright and horizontal positions. In our opinion, all these characteristics do indicate this exercise as a main candidate to explain the changes in the aforementioned variables.

In the present study, there was a significant increase in MIP and MEP after the yoga program. This effect may be an adaptation of the respiratory muscles to the breath training included in the program. The study carried out by Weiner et al. (2003) in elderly subjects suffering from chronic obstructive pulmonary disease provides evidence for this assumption. Those authors observed that the conditioning of abdominal muscles allowed the preservation of the width of the diaphragm muscles fibres and its ability to generate strength at the beginning of the contraction of inspiratory muscles, despite of the pulmonary hyperinflation. Some exercises of the yoga program matched the typical characteristics of respiratory muscle conditioning. This is the case of breath exercises that were adopted, such as Kapalabhati (sequence of forced exhalations) and Udiyana Bhandha (abdominal contraction after a forced exhalation), both performed with a static technique (to restrain contraction) and with a dynamic technique (several contractions with retention after the exhalation). Additionally, widening of inspiration musculature promotes the action of the expiratory muscle group which could justify the increase of MEP.

On the other hand, significant improvement of MIP, may have occurred due to the flexibility of the muscles responsible for inhalation, as previously observed (Teodori et al., 2003). Teodori et al.’s (2003) study investigated the use of global posture re-education (which included flexibility and respiratory exercises) and observed an improvement in the coordination of the action of that muscle group, probable due to the strength exertion required in the expiratory muscles during the execution of the appropriate posture.

## Conclusions

We concluded that a 12-week practice of yoga significantly improved the pulmonary function of elderly women. To our knowledge, this was the first study to report such adaptations in a western population of this subject group. These results draw attention to the possible importance of yoga practice in the elderly as a preventive exercise to impair age-related morbidity and improve the quality of life of these populations.

## Figures and Tables

**Table 1:** Body mass, resting heart rate and resting respiratory rate before (Pre) and after (Post) the experiment. Values are expressed as means and standard deviations

	**Yoga Group (n = 18)**		**Control Group (n = 18)**	
	**Pre**	**Post**	**Pre**	**Post**
Body mass (kg)	63.06 ± 13.4	63.11 ± 12.8	65.77 ± 9.4	67.58 ± 10.6
Heart rate (beat·min^−1^)	76.39 ± 8.03	74.61 ± 10.26**†**	77.28 ± 11.9	79.78 ± 12.09
Respiratory rate (cycle·min^−1^)	18.61 ± 3.15**†**	16.72 ± 3.12	14.61 ± 2.03	15.61 ± 2.59

† = significant difference between pre and post test in the YG (p≤ 0.05).

**Table 2:** Means and standard deviations of Tidal volume (V_T_), minute ventilation (V_E_), vital capacity (VC), maximal inspiratory pressure (MIP) and maximal expiratory pressure (MEP) in the two groups before (pre) and after (post) the experiment

	**Yoga Group (n = 18)**		**Control Group (n = 18)**	
	**Pre**	**Post**	**Pre**	**Post**
V_T_ (ml)	0–55 ± 0.22	0.64 ± 0.20**§**	0.56 ± 0.20	0.56 ± 0.11
V_E_ (ml)	9.19 ± 2.39	10.05 ± 2.11**†§**	7.92 ± 1.23	7.76 ± 1.09
VC (ml)	1.48 ± 0.45	2.03 ± 0.72 **†§**	1.96 ± 0.4	1.90 ± 0.43
MIP (cmH_2_O)	62.17 ± 14.77	73.06 ± 20.16**§**	70.94 ± 15.07	70.28 ± 15
MEP (cmH_2_O)	80.56 ± 23.94	86.39 ± 20.16**§**	75.83 ± 16.29	74.12 ± 16.02

† = significant difference in Post between the YG and CG (p≤ 0.05).

§ = significant difference between Pre and Post in the YG (p≤ 0.05).
